# The double burden of COVID-19 and Israeli military rule on persons with disabilities in the West Bank of the occupied Palestinian territory

**DOI:** 10.3389/fpsyg.2022.955828

**Published:** 2022-10-11

**Authors:** Suzan Mitwalli, Dina Kiwan, Lina Abdul-Samad, Rita Giacaman

**Affiliations:** ^1^Institute of Community and Public Health, West Bank, Birzeit University, Birzeit, Palestine; ^2^School of Education, University of Birmingham, Birmingham, United Kingdom

**Keywords:** COVID-19 pandemic, disability, occupied Palestinian territory (oPt), access to essential services, Israeli military occupation, checkpoints and barriers

## Abstract

**Background:**

The COVID-19 pandemic has enormous negative effects on people’s lives. People with disabilities (PWDs) who have been made vulnerable and who tend to face significant barriers in accessing public services are likely to be affected even more. This study aims to shed light on the impact of the COVID-19 pandemic on PWDs with special attention to their ability to access public services in the West Bank region of the occupied Palestinian territory.

**Methods:**

This qualitative study was conducted between March 2021 and October 2021. The study was divided into two phases. The first phase consisted of interviews with people with different disabilities, while the second phase targeted policymakers and stakeholders in discussion of the results of the first phase. A total of 16 interviews with people with different types of disabilities (7 males, 9 females) were conducted *via* telephone. For the second phase, a total of 6 interviews were conducted with stakeholders most responsible for addressing the issue of disability and the needs of PWDs in the West Bank. Analytical memos were prepared for all interviews. Main themes and subthemes were identified by reading and re-reading memos and transcripts until themes and subthemes emerged.

**Results:**

All participants agreed that the COVID-19 pandemic had affected all aspects of life for all groups of people, including PWDs. The effects of the pandemic exacerbated the overall living conditions and access to basic services for PWDs. The results show that access to public transportation, public services, and to health services was all interrupted during the pandemic. This was compounded by deterioration of the financial situation for PWDs which further worsened their access. The stakeholders’ interviews confirmed and explained the findings as mainly due to lack of prioritization of PWD’s rights and needs.

**Conclusion:**

The study emphasized that most of the reported barriers to accessing essential services were intensified during the pandemic. Furthermore, the results show that PWDs and their needs are not considered a priority by the Palestinian Authority, exacerbated by the Israeli occupation. Our findings underline the importance of including PWDs in any future crisis planning.

## Introduction

The COVID-19 pandemic has spread worldwide, affecting all populations, albeit disproportionately (Kuper et al., 2020; Lee and Kim, 2020; Shakespeare et al., 2021; Mitwalli et al., 2022). Making up more than 1 billion people worldwide, persons with disabilities (PWDs) (Kuper and Heydt, 2019) are among the hardest hit by the pandemic (United Nations, 2020). While PWDs are among the most impacted groups, they are also among the most excluded and vulnerable as they were generally absent from most of the COVID-19 response plans (Sabatello et al., 2020; Kubenz and Kiwan, 2021). This is not surprising as PWDs are often neglected in disasters and emergencies globally (Armitage and Nellums, 2020; Sabatello et al., 2020; Kubenz and Kiwan, 2021; Shakespeare et al., 2021). In Low-to-Middle-Income Countries (LMICs), where the majority of PWDs live (80%) (Banks et al., 2021), the strains of the pandemic and exclusion of PWDs in the COVID-19 intervention measures, are further intensified (Kubenz and Kiwan, 2021) as LMICs have weak infrastructures which are generally unable to respond and cope with such emergencies, have inadequate resources, and tend to lack social protection policies (Vieira et al., 2020; Kubenz and Kiwan, 2021; Shahzad et al., 2021).

Thus, the pandemic and its containment measures created extraordinary difficulties for PWDs, with varying difficulties depending on the type of disability. For example, disruption of daily routines, made it difficult for people with autism to cope with daily life because of sudden self-isolation (Willner et al., 2020). Lip reading is not possible for persons with hearing difficulties with the use of face masks and social distancing, making it especially difficult to obtain information on how to deal with the pandemic or how to access services (Shakespeare et al., 2021) among other problems. Using public transportation became a massive barrier to those with visual and motor disabilities due to the physical distancing requirement and the need to avoid touching surfaces because of the risk of transmission among especially persons with visual disabilities (Shakespeare et al., 2021). The sudden decreased access to rehabilitation and healthcare centers further exacerbated the physical health of those with motor disabilities (Lebrasseur et al., 2021). Moreover, it has been reported that social isolation disproportionately affects people with physical disabilities, resulting in poor mental health outcomes (Lebrasseur et al., 2021; Shakespeare et al., 2021). For persons with intellectual disabilities, the loss of social care from residential schools, day services, and other forms of care providers (Lebrasseur et al., 2021; Oviedo-Cáceres et al., 2021; Patel et al., 2021), increased the burden on families perhaps unaware of their needs or unable to provide adequate care (Jalali et al., 2020; Shakespeare et al., 2021).Thus, while containment measures to deal with the pandemic are deemed necessary (Bentley, 2020), the pandemic further heightened the risk of de-prioritization and exclusion of PWDs by the COVID-19 intervention measures (Kuper et al., 2020).

In the Israeli occupied Palestinian territory (oPt) (Amnesty International, 2022), Palestinians have and continue to suffer from the effects of the COVID-19 pandemic and its containment measures in ways which are similar to other populations in LMICs. Yet in addition, Palestinians including PWDs who form around 6% of the population of the oPt (Palestinian Central Bureau of Statistics, 2018), have also been enduring a double captivity: not only that of the pandemic, but also the chronic and ongoing Israeli military occupation of Palestinian land. On the West Bank, Israel is in full control of 60% of West Bank land, also known as Area C, and greatly limits the movement of people, resources, goods and livelihoods while inflicting both direct and indirect political and structural violence on the Palestinian population (Batniji et al., 2009). Additional human right violations include attacks by illegal Israeli settlers on Palestinian West Bank land, and on-going demolition of homes by the Israeli army which inflict further suffering on all of the population, including on Palestinian PWDs (United Nations Office for the Coordination of Humanitarian Affairs, 2020). During the pandemic, the Israeli military occupation army maintained its continuous violence against Palestinians and disrupted Palestinians efforts to contain the pandemic (Hamamra et al., 2021b). Adding insult to injury, PWDs also endure the neglect and de-prioritization of their needs by the Palestinian Authority (PA), which governs and has a measure of control over about 10% of the West Bank only (World Bank, 2010). It is necessary to note that the PA also endures serious Israeli occupation-related limitations as it lacks sovereignty over land which limits and curtails its ability to respond to crises, and largely depends on foreign aid (Giacaman et al., 2004). Prior to the pandemic, PWDs in the oPt were already enduring serious difficulties as many PWDs experienced poverty, high illiteracy levels and unemployment rates, with little opportunity to function and participate in society at the most basic levels (Mitwalli et al., 2022). These pre-COVID-19 systemic barriers to PWD functioning were critically aggravated by the pandemic. With the spread of COVID-19 to the West Bank beginning March 2020, and by May 2022, an estimated 657,573 people had been infected by the virus in the West Bank, with over 5,600 deaths recorded (Reuters, 2022) out of a population of 3.12 million (Palestinian Central Bureau of Statistics, 2018).

In the context of an already weak and Israeli controlled fragile West Bank economy and fragmented health system, the PA enforced a state of emergency with complete and partial lockdowns, closure of public spaces, and shifted school and university education to online teaching from March 2020 till the middle of 2021 to protect the population and contain the viral surge (Hamamra et al., 2021a; Mitwalli et al., 2022). This has had negative effects especially on Palestinian excluded groups, particularly PWDs, with life severely disrupted, access to services seriously curtailed, and poverty increasing among an already poor population. These circumstances prompted us to conduct an investigation focusing on PWDs and their families. The aim of this qualitative study was to provide insights into how the COVID-19 pandemic superimposed on pre-existing problems related to continued Israeli military rule and exposure to political violence among the West Bank population have affected PWDs who are among the most vulnerable groups in the population as they are suffering from particularly difficult circumstances and multiple barriers to a dignified and decent life. The study pays specific attention to health care access and selected public services, and the consequences of the pandemic and lockdown on economic life and livelihoods.

## Materials and methods

This qualitative study began at the end of March and was completed at the end of October 2021. Ethical approval was granted by the Institute of Community and Public Health, Birzeit University’s (ICPH/BZU) Research Ethics Committee. The study was divided into two phases. The first phase consisted of interviews with PWDs, while the second phase targeted policy makers and stakeholders in discussion of the results of the first phase.

We opted for individual interviews as opposed to focus group discussions especially with policymakers/stakeholders. This is because our experience indicates that when discussing contentious issues related to unmet needs of priority groups which are neglected, focus groups can induce arguments motivated by defense, denial or justification of why things are the way they are, rather than a discussion of issues and how to solve them. In individual interviews, participants are inclined to be reflective rather than defensive, and are more willing to acknowledge problems without losing face.

The ICPH research team consisted of two researchers and one assistant. The team employed two contact facilitators to help in recruiting potential PWDs interviewees. The first list came from the Community Based Rehabilitation (CBR) Program and the second came from an activist with a disability. In both cases, potential interviewees provided initial verbal consent to participate in the study. During this time period, interview questions were developed in discussion among the three-person team, and in line with the aims and objectives of the study. The interview schedule was piloted and modified to correspond to the findings of the pilot.

Interviews with PWDs or their mothers were conducted in May, June and July 2021. Due to the constraints of the COVID-19 pandemic, all interviews were conducted *via* telephone, as internet access may have been inaccessible to all participants. Each interviewee was contacted by phone and verbal consent was obtained orally and noted in writing. This included the consent to record the interview. Each interview was audio recorded and saved on a password-protected computer. There were two interviewers: the lead interviewer responsible for probing and a second interviewer who was mainly in charge of note-taking but also engaged in the questions and probing when needed.

The initial aim was to interview 16 participants from the list of 25 provided by facilitators. PWDs with motor disabilities dominated the lists, with fewest interviewees being PWDs with hearing disabilities. Our purposive sampling procedure included variables related to region (North, Central, and South West Bank governorates), geographic area (urban, rural, or Palestinian refugee camp), gender, and age. These key variables were accounted for when choosing participants to ensure diversity and a spread of experiences. Out of this list of 16 potential interviewees that we had selected, there were two refusals despite providing initial consent to facilitators. These refusals were replaced with other potential participants from the lists we obtained from facilitators.

Of the total 16 interviews that were conducted, 6 were completed with participants with a motor disability, 4 with an intellectual disability, 3 with a visual disability, and 3 with a hearing disability. For participants younger than 18 years old and for children with intellectual disabilities, the mothers were interviewed either alongside the participant or alone. Additionally, a mother of a participant with a hearing disability was interviewed as a sign language translator was not available in the north of the West Bank. The interview questions focused on the barriers faced by the interviewees mainly during the pandemic in accessing different services including health and public services in general. We also asked about changes in the economic status of the family during the pandemic.

The second phase of this study in October 2021 consisted of interviewing local, national and international policymakers. The aim of these interviews was to create a dialogue centered around the results of the interviews with PWDs or mothers of PWDs. Interviews included presenting the summarized findings from the PWD interviews to these policymakers; finding out whether they endorsed the findings; and obtaining their views regarding why the PWD’s identified needs were not being met, especially during the ongoing COVID-19 pandemic.

A total of six interviews were conducted with one representative from a local non-governmental organization, three representatives from the Ministry of Health (MoH), Ministry of Education and Ministry of Social Development (MoSD) each, and two representatives from international organizations; United Nations International Children’s Emergency Fund (UNICEF) and Diakonia, both are very active in disability rehabilitation in the country. Each interviewee was contacted by phone and verbal consent was obtained orally and noted in writing. This included the consent to record the interview. All but one interview was conducted *via* Zoom. The remaining interview was conducted face-to-face. Interviews were audio recorded and stored on a password-encrypted computer held in the lead interviewer’s office.

Immediately following all interviews, analytical memos were composed based on the written interview notes, and then finalized by listening to the interview audio recordings and fine tuning content. Main themes and subthemes were identified by reading and re-reading memos and transcripts until emerging themes and subthemes were agreed upon by the research team members.

## Results

Almost all PWDs participants agreed that the COVID-19 pandemic had affected all aspects of life for all groups of people, yet with more intensity for people with disabilities (PWDs). The effects of the pandemic exacerbated the overall living conditions and access to basic services for PWDs, as a 20-year-old male participant with a motor disability from a northern West Bank (WB) village stated:

“With the pandemic which has become a burden on people, can you think of how this would be for people with disabilities…? The Corona pandemic affected all sectors (of society) psychologically, socially, health wise and economically.”

In addition to the difficulties PWDs participants faced during the pandemic, they were keen to point out selected and important chronic barriers to accessing a variety of services they even endured before the pandemic. Elaborating on pre-pandemic difficulties by PWDs, participants helped explain how their access to services had deteriorated even more during the pandemic. In this respect, the stakeholder interviewees confirmed that most of PWDs’ difficulties were related to the pre-pandemic period but with more severity during the pandemic. Moreover, they agreed that PWDs’ needs were not considered during the pandemic even though they were amongst the most affected groups by the pandemic. One related issue according to a stakeholder participant is the lack of representation of PWD organizations in the emergency committees. She noted:

“There were no institutions of PWDs in emergency committees to take decisions (relevant to the needs of PWDs). No one felt the problem, they were not seeing it, and this indicates a defect. No one talks about the needs of PWDs without them being there.”

### Barriers to access public transportation

Public transportation was one of the key issues that most PWDs participants struggled with during the pandemic, as it was either disrupted partially or completely. The PWD interviewees acknowledged that this barrier was also for all people and not specifically for them. The disruption was due to different lockdown requirements enforced on people and private and public transportation vehicles. For example, most PWDs interviewees noted the placement of Palestinian police checkpoints at city entrances. This lockdown restriction hindered all movement completely, which barred access to key services. A 66-year-old mother of 8 children with intellectual disabilities living in a southern West Bank village noted:

“By God I took a risk once, and the police was following me. I went to bring medications for my children as they (the police) were following me. I told them that I want to take medications for my children, I took a risk for my children. Yes *wallahi* (by god), they were about to send me back but when they saw the children’s cards they agreed (to let me go). *Wallahi* I faced difficulty and fear too, fear.”

Another female 30-year-old participant with a hearing disability from a central West Bank village elaborated:

“A burden on us, the police did not believe that we were going for a health issue, they thought we were going for an outing. The police did not deal with us well, they were not convinced (did not understand) about the situation of people. Our village is not part of the Palestinian Authority (it is in Area C which is completely controlled by Israel) so when we would enter the Palestinian Authority areas there were difficulties.”

Elaborating on the transportation-related difficulties in accessing his university during the pandemic, a 20-year-old male participant with a motor disability from a northern West Bank village noted:

“Imagine during the pandemic period, one wants to go to an educational institution, one needs to think how to go and move. You have transport (which is) much harder in the Corona. So he will think of movement before he thinks of education. So here you have deprived him of more than one right. You deprived him of education. You deprived him of the right to movement. You deprived him of independence…. this was (the case) before Corona but when Corona came life became harder.”

This example illustrates the multiple and intersecting impacts on PWDs where both rights to education and rights to free movement are impacted.

Similarly, two participants with visual disabilities reported difficulty in accessing public transportation to reach their universities. For example, a male 20-year-old participant living in a rural area of northern West Bank elaborated on the increased challenge to access the university to buy past papers, textbooks, and paying tuition fees since the public transportation to the university was very limited because of university closures due to the pandemic.

Additionally, the increased cost of transport due to the pandemic related restrictions prevented some participants at times from accessing services, especially those living in rural and camp areas as this 18-year-old female participant with a hearing disability from a camp in the center of the West Bank emphasized:

“The difficulty of transport and payments during Corona. Thirty five Shekels (the costs) were not available…the difficulty of getting there, the difficulty of transport to the hospital from a financial point of view.”

Another male participant aged 33 years old with a motor disability from an urban area of the south West Bank noted:

“It is difficult for a person with disability to ensure transportation, financially difficult in ensuring transport, and who is in a wheelchair without a private car (must pay) 100 Israeli Shekel for the smallest trip.”

In line with the reported problems by PWDs participants in accessing public transportation, almost all stakeholders’ interviewees agreed that public transportation has always been a challenge, but it became increasingly more difficult during the pandemic. A participant from MoSD maintained that the transportation problem is complex and there is a lack of cooperation from taxi drivers. He stated that drivers do not consider that PWDs require more time to get into the vehicle, and emphasized that this corroborates PWDs’ demand to obtain special transportation services for them.

It is worth mentioning that the PWDs participants also reported lack of accessibility to public transportation as one of the main barriers that always exists even in the pre-pandemic period, particularly for people with visual and motor disabilities. It was explained that those with visual and motor disabilities depended greatly on the assistance of others to access transport due to the lack of adaptation of the public transport system to accommodate, for example, wheelchairs, let alone auditory sign and public transport stops, given that there are hardly busses going around and that shared taxis seem to stop anywhere wherever there is a potential customer waving their hands indicating they need a ride.

### Barriers to access public services

Another barrier reported by most PWDs participants, and confirmed by stakeholders, was related to accessing public services which has always been a difficulty for PWDS in the oPt, given that public buildings are not adapted for people with different types of disabilities. The interviewed stakeholders admitted that some health facilities are partially adapted for people with partial motor disabilities, but they are not adapted for other kinds of disabilities such as those with visual and hearing disabilities. This young 20-year-old male participant with a visual disability from a northern West Bank village explained it:

“Adaptation (to the needs of PWDs) for daily life is not provided (by ministry or government) unfortunately, such as (in the case of) streets, public areas and public institutions. Buildings are not adapted for people with disability, no elevators adapted to Braille. For people with physical disability no rail; for those who have a hearing disability, there are no sign language translators.”

During the pandemic, difficulties in accessing public services were exacerbated by the fact that service providing institutions were working only partially during the lockdown, which presented a challenge for PWDs. Most PWDs interviewees reported problems such as limited opening hours, a partial number of operating employees, and long waiting time resulting sometimes in multiple visits to obtain the needed service. A female participant with a visual disability aged 20 from a northern West Bank city noted:

“The conditions of closure presented challenges depending on where one wanted to go. For example if one had a trip (needed to go to) to the bank, had a trip to the Jawwal company (mobile phone company), a trip anywhere and wanted to go during a particular time and there was closure, things would get obstructed for him/her.”

Local and international NGOs play an important role in providing activities for PWDs. During the pandemic they were closed or canceled their activities. In this regard, a 21-year-old male interviewee with a motor disability from a central West Bank camp shared his frustration at no longer being able to participate in trainings.

Some interviewees pointed to the difficulties in accessing recreational facilities during the pandemic. This was reported mainly by the mothers of persons with intellectual disabilities. This has negatively affected the physical and mental health of people with intellectual disabilities and their opportunity to get out of the house as this mother of a daughter, aged 32 years, with intellectual disability, from a central West Bank city, revealed:

“My daughter’s mental well-being has become very bad, and she started wetting herself and keeping things in her heart (internalizing things) and she gained weight and her body became bloated because she needs to walk, and she began to take a diuretic. The public parks are closed and people are not allowed to shake hands, where do I take her?”

And this mother of 10 years old boy with autism from a rural area in the north of the West Bank told us:

“Corona affected my son, no activities, no clubs or swimming and public parks closed and he likes such things, he was imprisoned at home sitting with the TV (on) and phone all the time and eating, he gained weight.”

A male 33-year-old participant with a motor disability from a southern West Bank city also emphasized that confinement at home and the closure made him distressed. He noted:

“Closure created psychological pressure, created a psychological situation. That is, one stays at home with no breather. One goes out. the shops are closed, no income, this played a (negative) psychological role.”

In addition to the above-mentioned barriers in accessing public services and the related consequences, lockdown and social distancing requirements created a challenge for PWDs who needed personal assistance. This was especially for people with hearing disabilities and people with visual disabilities, who usually rely on personal assistants due to inaccessibility difficulties. The problem was that having personal assistants during COVID-19 increased the risk of contracting the virus.

This need for personal assistance has always been an issue for people with hearing disabilities when they seek public services even before the pandemic. For example, the absence of sign language translators at banks which created a problem for privacy and independence as a female participant with a hearing disability stated that she doesn’t want anyone to know how much money she has, and this is not possible due to lack of privacy resulting from the unavailability of translators in banks.

The absence of sign language translators in all services was also noted by the stakeholder interviewees. The interviewee from MoSD reported that the Ministry requested banks to provide sign language translators and to have a Braille system available for banking services. According to him, the law does not prevent a person with a disability to have full autonomy regarding their banking services and thus there is no need for a guardian. However, this request has to yet be implemented.

The difficulty of maintaining social distancing requirement during the pandemic was also a problem discussed by interviewees with motor disabilities. For example, a male participant maintained that keeping social distance was difficult for him as a physically disabled person who uses a wheelchair. He explained that his friends push his wheelchair and this goes against social distancing guidelines. Another 21 years old male participant with a motor disability from a central West Bank camp noted:

“Maintaining the required distance as a movement PWD was difficult for me.”

For accessing public health information related to the pandemic and governmental restriction measures, most interviewees explained that they relied on several types of media. These media included television, social media platforms, government daily briefing reports, and Palestinian MoH instructions. Sometimes, participants depended on their family members to deliver updated knowledge. For example, a female interviewee with a hearing disability particularly complained about no longer having a sign language translator on the national TV Channel. Prior to the pandemic, there used to be a sign language translator for one of the news broadcasts channels. However, this was interrupted during the pandemic. And this caused the participant to rely on her mother to update her on the daily news broadcasts.

### Barriers to access to health services

In general, PWDs participants who required health services during the pandemic emphasized the challenges that they had faced. For those who did not need such services, they referred to experiences of others whom they knew had struggled to reach such services. Some of the obstacles in accessing health services were shared among different types of disabilities.

Some stakeholders explained the challenges PWDs faced in accessing health services and lack of prioritization of their health needs, partly due to the Palestinian Authority (PA) focus on pandemic related precautionary measures which negatively affected health services, as one interviewee from MoH noted:

“Preventive measures to control the spread of the virus affected health services in remote areas and those most affected were PWDs; they were an excluded group and they became a neglected group during Corona.”

Almost all the interviewees with disabilities and mothers of PWDs believed that PWDs have low immunity and this could predispose them to catching the COVID-19 virus. Thus, some participants feared accessing health services to avoid catching the virus. One mother of a 16-year-old daughter from southern West Bank camp explained:

“During the Corona I kept her (her daughter) at home, and would go out and bring her necessities such as urine bags. I install for her an internal urine bag and I change it every month. I used to take her normally to the hospital, but was afraid and protected her because my daughter does not have immunity. I placed a mask on her, and dressed her with a burnus (covering all the body), and sanitized. I suffered during corona with my daughter but without making her feel this.”

In addition, the restrictive preventive measures related to the pandemic such as mandatory face-masks when entering the health centers, reduced staff availability and limited working hours were a problem for some participants, as a male participant with a motor disability elaborated on this issue and explained that he either delays accessing the public clinic due to crowding or he goes to a private clinic where he needs to pay for the service.

Another main obstacle even before the pandemic, reported by almost all PWDs participated in the study, was the lack of essential medications at the government health clinics. During the pandemic, this problem was exacerbated even more as it also coincided with physicians’ strikes at the time. The lack of medications forced some participants to buy them at their own expense, despite the difficult financial situation during the pandemic. As a 50 years old female participant with a motor disability from a northern West Bank village noted:

“The lack of medication was there before corona, but after corona it increased. Sometimes there were no medications at all, or not all medications were available, so you go and find one or two kinds of medication (only). Maybe because of closures and strikes, they said as long as there is a clinic strike, we are forced to buy (medications) from outside (the clinic, privately).”

In this regard, all of our stakeholders interviewed agreed that the consistent lack of necessary medications is a chronic problem. The participant from MoH elaborated that the ministry has debt for the local pharmaceutical companies, which contributes to the inconsistent supply of certain medications.

For PWDs, participants who reported having been infected with COVID-19, they reported that they had to purchase the needed medications for treatment. A 33-year-old male participant with a motor disability from a southern West Bank city also complained that the process of obtaining some medications related to COVID-19 was confusing for him and his wife who also has a motor disability. He explained that there were conflicting referrals by the employees at the government clinics which confused them in terms of where to seek the service, and that they faced difficulty in obtaining some medications from the clinic. He also recommended that the government should provide oxygen-making machines to COVID-19 patients with disabilities as they have difficulty in accessing health services.

Related to the issue of PWDs who were infected at the beginning of the pandemic and were kept in the quarantine centers, some stakeholder participants referred to a challenge they faced in being away from their families especially for those who have hearing or severe intellectual disabilities and autistic persons. This was because they needed to be cared for by family members. Moreover, the staff in the quarantines were not qualified on how to communicate with PWDs and had scarce knowledge about their needs, let alone how to respond to these needs.

Some PWDs participants viewed the closure of outpatient clinics at hospitals and the conversion of hospitals to accommodate the COVID-19 patients’ cases only as main barriers in accessing health services. This affected those who were in need of regular medical follow-up, and had disrupted the provision of routine health services and delayed medical operations, as some participants reported. A mother of a 32-year-old daughter with intellectual disability from central West Bank city stated:

“My daughter needs an MR picture and she had an appointment on the 5th of October. But they told me entry to the hospital is banned and told me to do it at my expense… I do not have 3000 Israeli Shekel to make it at my expense.”

Two interviewees, one with a hearing disability and the other is a mother of a person with hearing disability, elaborated on their frustration over the same problem of repeated delay of required surgeries, in addition to the long process and waiting time usually related to the surgeries at the governmental hospitals. An 18-year-old female participant with a hearing disability from a central West Bank camp mentioned:

“Difficulty of reaching (health services) during the pandemic because (government) outpatient clinics do not receive (patients) every day. They were closed on many days especially during corona. And there was a strike so they (clinics) did not operate, and no surgeries as before. They gave priority to corona patients and emergency. The postponement (of my procedure) puffed my eyes up and affected my vision and affects breathing.”

Concerning other impediments related to outpatient clinics closures, an interviewee with visual disability discussed his experience in seeking his impairment rating report when he needed it during the pandemic. He faced much difficulty in reaching the medical committee at the hospital as he had to go through multiple visits and a long period to reach the committee and obtain the report.

Our MoH interviewee agreed that closures of outpatient clinics created problems for PWDs. He reported that the ministry was forced to take strict precautionary measures to stop the spread of the COVID-19 virus. This was because hospitals were a possible place of infection. The participant from MoSD stated that the effect of this closure affected PWDs more than other groups. He noted:

“Closing outpatient clinics affected all people but PWDs were affected more, their rights were violated twice, once as PWDs and another because of Corona.”

Additionally, the lockdown restrictions also shifted the medical follow-up routine. One female interviewee with a motor disability from a northern West Bank village, who suffers from a chronic disease decided to access the clinic close to her rural residence rather than accessing the more advanced clinic with better services due to the restrictions. This participant avoided accessing a specialist doctor in the city when she broke her leg due to the closure and to reduce costs. Instead, she approached a doctor in her village which resulted in deteriorating effects on her health. She noted:

“I used to attend that (Governmental) clinic every month. When corona happened I stopped going. I take my medications from another clinic. At that other (governmental) clinic (I used to go to before corona) they have more specialized doctor and modern laboratory equipment than the one I go to during corona.”

Almost all PWDs participants or their mothers stated that they were neither approached nor informed by the MoH about COVID-19 vaccines. Most stakeholders’ interviewees confirmed that PWDs are not considered a priority by the government at all levels including COVID-19 vaccination campaigns, and pointed to the lack of policies that can support them. Our MoH interviewee noted that vaccination centers were mainly located in the cities at the onset of the vaccination campaign. According to him, the MoH should have also considered providing the vaccine at primary health care clinics located in the villages. In this way, vaccines would have been accessible to people who cannot access the cities easily, including PWDs. He also asserted that a PWD database to include names and home addresses should be available for the Ministry to reach PWDs who could not reach the clinics to get their vaccination, and he suggested providing vaccination services by way of mobile clinics. De-prioritization of PWDs is represented also by the lack of disaggregated data about PWDs who were infected by the COVID-19 virus which should be available at the MoH according to a stakeholder participant from an international organization.

In addition to these pandemic-related obstacles, participants with all types of disabilities stressed that some barriers they face are structural in nature and not related to the pandemic, for example, problems with the national health insurance coverage.

In response to this, a participant from the MoH mentioned that the pandemic delayed the promulgation of a new disability law and preparation of the implementing regulations. This new law would greatly improve the situation for PWDs as many of our participants (both PWDs and stakeholders) confirmed. However, an interviewee from MoSD claimed that the high cost of implementing the law is the reason for the delay:

“The PWD Law (new) has been with the Council of Ministers and has not been approved till now, and this is unjustified.”

### COVID-19 related problems accessing health services by type of disability

While there were common problems reported by our PWDs participants regarding accessing the health services, there were also some varying difficulties experienced by participants based on the type of disability they had. The interviewees who have motor disabilities reported specific problems related to their disability including the closure of the rehabilitation centers due to the lockdown, and interruption of the rehabilitation follow up. A male 21-year-old participant from a central West Bank camp noted:

“Rehabilitation centers closed down during the corona pandemic, and they are the centers that help people with disabilities most or provide them with treatment, whether physical therapy or assistive devices or any other activity. They are the only ‘breather’ for some of the people with disabilities, so they should have found an alternative plan or anything so that they should not close… it is coming back partially, but still (there is) fear or (those responsible) do not know how to prepare for going back (to work).”

Moreover, some of the interviewed participants with motor disabilities agreed that the cost of rehabilitation sessions was the main barrier even during pre-pandemic times, in addition to the cost of transport to access these centers. Fearing to contract COVID-19 during the visits was also a concern.

Two participants with motor disabilities elaborated on the difficulty of obtaining mobility devices during the pandemic. This difficulty in acquiring mobility aids was due to the closure of the rehabilitation centers. Another problem raised was the shortage of hygiene supplies such as diapers and urinary bags that were costly and scarce during the lockdown, as recalled by a mother of a 16- year-old girl with a motor disability from Southern West Bank camp.

Participants with hearing difficulties stressed their need to get new hearing aids and reported that closure during the pandemic further delayed obtaining them. This was because the hearing aids were not available at the centers as they were mostly closed due to the lockdown. Also, the cost of the hearing aids was a barrier both prior and during the pandemic. Another related problem is the inability to get new batteries for the hearing aids again because of the centers’ closure and inaccessibility problems. Some stakeholders confirmed what these participants reported, and emphasized that there was a shortage of hearing aids and the devices’ batteries, and they were only available in the main centers located in the cities.

This is reflected in the following quotes by participants with hearing difficulties:

An 18-year-old female participant from a central West Bank camp noted:

“The situation was difficult because hearing aids do not reach centers because there are no hearing aids at the crossing and because of tax, nothing gets in.”

And this 30-year-old female participant from a central West Bank village added:

“Hearing disability needs batteries and hearing aids, and they were not available during the pandemic. Also the services institutions may give them once or twice for free and for minimal fees. I need more than once or twice.”

Regarding the difficulties persons with intellectual disabilities encounter, mothers of persons with intellectual disabilities reported the general need for day-care centers for their adult children as the main unmet need. A mother with 8 children with intellectual disabilities and two out of the eight also with motor disabilities emphasized the negative effect of the pandemic regarding the closure of the day-care centers during the pandemic. Her two sons with multiple disabilities used to attend such centers before their closure. This added greater burden on the mother to take care of these two sons who require more care, particularly in terms of bathing and other hygiene tasks. One of the mothers planned to send her daughter to a day-care center just before the pandemic and started doing so but the center closed due to the pandemic. The same need was reported by a mother who has three children with intellectual disabilities and who has always been asking for day-care centers that are not available in her area prior to the pandemic.

Our stakeholders’ participants confirmed that the closure of the centers for people with severe intellectual disabilities impacted the PWDs and their families who were unequipped to respond to their needs. According to a stakeholder participant, this had mostly affected the mothers of persons with intellectual disabilities who prefer that their children stay in the centers. She stated that:

“Closure of centers was a problem for families although I see it as a double -edged sword. Some families had not seen their children for a while, and this (closure) was a burden because they are not used to it, and mothers preferred that their children would stay at the centers. However, I also find that people with mental disabilities have a right to be in their families’ homes.”

### COVID-19 related economic barriers

In addition to the difficulties related to accessing different services, all PWDs interviewees agreed that the most notable effect of the pandemic was the deterioration of the economic situation for all groups of people, and PWDs were among the most impacted of groups, as this 20-year-old female participant with a visual disability from a northern West Bank city stated:

“The pandemic affected the economic situation of society in its entirety, from merchants, to daily workers. The salary was enough (for our family) before, and the two of us were studying at university… (now) material (money) is less and work is less, and my family cannot educate (pay the cost of education) of more than one (person).”

Or as this 21-year-old male respondent with a motor disability from a central West Bank camp explained:

“Most were affected, and those who had a job lost it and whoever had a material resource (income) almost disappeared.”

However, almost all PWDs participants already had stressful financial situations prior to the pandemic as the following participants explained:

A mother of 18-year-old son with a hearing disability from a northern West Bank village explained it: “My life is all Corona (in bad financial situation), not a year or two.”

And this 18-year-old female participant with a hearing disability, who reported:

“The economic condition was not affected (by the pandemic). It was bad from the beginning (before the pandemic)”

Despite the financial crisis PWDs were experiencing during the pandemic, most participants did not receive significant financial aid from the Palestinian Authority. Some participants complained that they were already excluded from MoSD monthly stipends prior to the COVID-19 pandemic. This exclusion was due to MoSD’s social needs assessment, as some PWDs were not considered in need of financial aid, and as a young interviewee with a motor disability maintained:

“I tried to contact MoSD to give me some money but it did not respond, they tightened their conditions (for financial support) and they are harsh. I went to the responsible person and she told me that I do not need help, one must see a destroyed home or a broken wall to give you help even though you fulfil the requirements (for providing help). The material situation is hard and I have a disability (which qualifies me for financial help from the MoSD) and support a family of seven, and this requires that they provide me with a salary. I talked with the Union (of PWDs) and they tried (to discuss with the MoSD) without result.”

Even PWDs interviewees who reported receiving a monthly stipend from the MoSD maintained that the stipend is insufficient as this mother of 10-year-old autistic son revealed:

“We benefit from the MoSD, even though this benefit is the shadow of shadow (very little benefit).”

Additionally, the participants reported a consistent delay in receiving their stipends. The payment is scheduled to reach participants every 3 months. Instead, it is usually received every 4 months. This was always the case prior to the pandemic and was exacerbated during the pandemic as this 33-year-old male participant with a motor disability elaborated:

“The MoSD gives me 750 Israeli Shekels every three months and sometimes every four months. (But) they do not abide by the timing (of payments) sometimes it takes (them) 5 months (to send payments).”

All stakeholders interviewed agreed that the financial situation of PWDs and their families deteriorated during the pandemic. They also confirmed that there is a consistent delay in MoSD stipends and particularly during the pandemic. The interviewee from MoSD reported that beneficiaries are also not reimbursed for the unpaid stipends. This is due to the financial crisis of the PA in general as some participants reported, and in particular during the pandemic because of PA’s focus on the pandemic related costs such as costs of COVID-19 tests and treatment at hospitals as an interviewee from MoH reported.

Surprisingly, PWDs who benefit from the MoSD monthly stipends were excluded from the COVID-19 related governmental limited financial aid launched during the first year of the pandemic under the title of the *Waqfet Izz* or “Dignity Stand”: A mother participant of 8 children with intellectual disabilities noted:

“I was infected with Corona and I have diabetes …. And got sick with corona and all my family got sick with corona… money (was given) to those with corona but us no, we (PWDs) are among the people who are forgotten.”

The stakeholder interviewees explained that COVID-19 related governmental aid was directed toward people who lost their jobs during the pandemic, mainly workers on a daily basis, and PWDs were excluded from this aid.

Informal financial aid to PWDs, described by PWDs interviewees as aid received from people here and there who support PWDs sometimes but as persons, not institutions, was also negatively affected since the pandemic broke. Most interviewees reported that such aid was cut because all people were affected financially. They only received small amounts of financial aid or in-kind aid mostly as food baskets during Ramadan (the holy fasting month for Muslims):

This mother participant with 3 children with intellectual disabilities explained:

“Now no one looks up anyone (meaning helps) because everyone sat (at home) with Corona and did not work, so whoever wanted to give you (money or support), brings it to himself and home (instead).”

This financial hardship caused some PWDs and/or their families to live in debt as this 18-year-old female participant with a hearing disability revealed:

“When debts are piled up the shop owner demands (that we pay), and sometimes, they scream at my mother. Once the shop owner exposed and disgraced us to the world when my mother took on debt washing soap and he came and made a wedding at home (meaning figuratively lots of noise so everyone heard and the family was exposed and disgraced). And my mother was very upset and sad.”

While access to public transportation, public and health services was constrained as elaborated above, the financial hardship PWDs were experiencing during the pandemic further hindered them from accessing these services and made the access even more difficult.

### Israeli occupation barriers

In addition to all services access difficulties, some participants elaborated on the barriers they faced in both the pre- and during the pandemic period because of Israeli occupation of Palestinian land and specific violation measures and human rights abuses, especially concerning their ability to seek medical care. This includes difficulty in obtaining permits to enter Israel, including illegally Israeli annexed East Jerusalem where many of the Palestinian specialized services are located; and exposure to tear gas at Israeli army checkpoints, difficulties in traveling abroad (either need for permits or having to deal with checkpoints and crossing difficulties), and lack of qualified medical staff locally, yet travel restrictions barring access. A male 20-year-old participant with a motor disability from a northern West Bank village stated:

“You (Palestinians) have no competencies because of (Israeli) occupation. There is no high-level medical treatment and for precise things (procedures). How many cases of disability because of occupation… you live while you are not allowed everything, and your movement is not easy, going and coming back (because of Israeli occupation, checkpoints, and restrictions of movement from one place to another and also out of the country).”

Another male 21-year-old participant with a motor disability from a central West Bank camp elaborated on the problem of requiring permits from Israel to be able to get to hospitals inside Israel, given the lack of services in the West Bank:

“Things used to happen before corona, things which are difficult to describe. I used to have follow ups at hospitals inside (Israel), and after preparing all things (requirements) from MoH and UNRWA, and preparing all papers comes the permit request. You request a permit (to enter Israel) and they (Israel) delay to the point that you postpone your appointment more than once.”

Regarding the barriers our participants faced due to Israeli occupation during the pandemic, a few participants reported no direct effect on the personal level. However, these participants recognized occupation’s impact (on the nation, society) in general as this mother of a daughter with intellectual disability maintained:

“The biggest enemy for us (Palestinians) is (Israeli) occupation, and Corona topped it on us, it became an extra (burden).”

Other participants, especially those who live in Palestinian refugee camps reported experiencing direct Israeli violence such as being exposed to invasions (the Israeli army going into camps, firing in the air, searching homes, arresting people, firing at people, tear gas etc.) even during the pandemic, as one male 21-year-old interviewee with a motor disability from a central West Bank camp reported:

“During the Corona pandemic, invasions are continuous as well as arrests here in the camp. They would shoot, invade during the day. All throughout, the (Israeli) occupation (army) invaded the camp at night, after midnight. During the Corona pandemic they attack during the day for long hours. You leave the lecture (on zoom) and you observe what happens, and you become afraid for those around you, and this affects the psychology and the economic situation and work.”

Or as this respondent, a mother of a 16-year-old daughter with a motor disability from a southern West Bank camp reported:

“When my daughter used to go to the center and they (Israeli occupation forces) would return the bus (denied entry) my daughter would cry and cry… I used to go out when they showered tear gas and get her siblings to go out to bring her because I am afraid for her from the gas.”

Moreover, a mother of a child with a hearing disability does not allow her son to visit his married sister in another city in the West Bank by himself. This is because she worries that he could be shot at the checkpoint, since he has a hearing disability and may not comply with the Israeli soldiers’ orders. The mother provided an example of how one of her son’s friends was killed at a checkpoint during the First Intifada (uprising) because the soldiers called to him and he did not respond. For this reason, she taught the international sign of “I’m deaf” and sign this whenever they travel between the oPt and Jordan. She stated:

“When we used to travel to Jordan, he would sign from far to the Israeli at the window (security), I am deaf, he would spread his hand and place two of his fingers on his mouth and then raise them onto his hears. This is the international sign, that I am deaf.”

## Discussion

This study provides an overview of the experiences of PWDs living under the Israeli military occupation in the West Bank of the oPt in accessing services during the COVID-19 pandemic. It is clear that most of the reported barriers to accessing essential services and public spaces which are needed by PWDs in order to attain a decent and dignified life existed in the period before the pandemic began. However, the findings stress that most of these problems were intensified during the pandemic among PWDs. Such findings were also noted by Hamamra (2022) focusing on other vulnerable groups such as women, where it was found that domestic violence against women in the oPt increased during the pandemic as well. Our study also contributes insights to the international literature by confirming that PWDs in the oPt experienced most of the impediments faced by persons with disabilities in the LMIC in accessing essential services during the pandemic. It also points to the compounded negative effects of the pandemic and warlike conditions on vulnerable groups in general and on PWDs in particular.

In addition, our interviewees emphasized the lack of prioritization of PWDs during the pandemic by the Palestinian Authority, which they thought could be partly explained due to the lack of political will and shortage of budget to issue and implement laws related to PWDs. Various studies have confirmed that PWDs’ access to different services was already limited pre COVID-19 and have been worsened during the pandemic (Diba and Zakaria, 2020; Lebrasseur et al., 2021; Rathore and Qureshi, 2021).

Our study shows that access to public transportation, public services, and health services were all interrupted during the pandemic. Difficulties to access public transportation were a result of lockdown requirements which prohibited public transportation and restricted PWDs from accessing key services. This result is in line with what Cochran (2020) and Lebrasseur et al. (2021) found, with a greater challenge reported for persons with motor disabilities and persons with visual disabilities (Oviedo-Cáceres et al., 2021), as also the case is in our study.

Additionally, access to public services was a crucial issue highlighted by our study participants. Their concerns focused on accessing service-providing institutions that were working partially during the pandemic, which in turn introduced a challenge for PWDs, who then needed multiple visits to obtain the services. More specifically, closure of recreational facilities was considered a major difficulty by our interviewees who are mothers of persons with intellectual disabilities. This was due to the direct effect on the physical and mental health of their children which is also emphasized in the literature (Courtenay and Perera, 2020; Al-Zboon, 2022). Study participants with hearing and speech disabilities and visual disabilities shared their particular difficulties in accessing public services due to their reliance on personal assistance that went against the social distancing restrictions of the Covid-19 pandemic. In line with this, some persons with motor disabilities echoed the same concern in facing difficulties in keeping a social distance when they need mobility help. Rathore and Qureshi (2021), along with Cochran (2020) pointed out this challenge and explained that persons with disabilities and their helpers might be at risk of contracting the COVID-19 virus given the close contact.

In terms of accessing public health information related to the pandemic and governmental measures, our participants have not experienced accessibility challenges except for persons with hearing disabilities who relied on their family members. This is similar to a study in which children with hearing disabilities and intellectual disabilities depended on their parents to receive the information in an accessible way. Some other studies found that PWDs faced major difficulties in accessing pandemic related information in an accessible and inclusive way, especially for people with visual disabilities and hearing disabilities (Gopinath et al., 2012; Kuper et al., 2020; Samaila et al., 2020; Vieira et al., 2020; Oviedo-Cáceres et al., 2021).

Access to health services was one of the most challenging issues for our participants as found elsewhere (Diba and Zakaria, 2020; Perera et al., 2020; Samaila et al., 2020; Rathore and Qureshi, 2021). The government’s focus on precautionary measures to combat the pandemic was considered a major reason for the lack of access according to our study stakeholders, as also indicated in studies conducted in Nigeria and Pakistan (Samaila et al., 2020; Rathore and Qureshi, 2021). Additionally, the majority of PWDs participants thought that their immunity is weak, which was for some participants a reason to avoid accessing health services, fearing to catch COVID-19 virus (Lebrasseur et al., 2021).

The barriers to access health services were multiple based on our study findings. The closure of outpatient clinics at the governmental hospitals during the pandemic was a main difficulty facing PWDs, who seek their routine healthcare follow up; a challenge also emphasized in other settings (Armitage and Nellums, 2020; Perera et al., 2020; Samaila et al., 2020; Rathore and Qureshi, 2021). Delay in scheduling medical operations and tests in addition to the shift of medical follow-up routine impacted the health of some participants. According to Perera et al. (2020) and Samaila et al. (2020) some PWDs already have pre-existing health needs both not specific and specific to their disabilities which explain the adverse effect on the health of PWDs. Furthermore, the shortage of essential medications at the primary health clinics during the pandemic and oftentimes pre-pandemic was another obstacle, also noted by Banks et al. (2021), Lebrasseur et al. (2021), and Rathore and Qureshi (2021).

Specific health services-related difficulties by types of disabilities were also reported in our study. People with motor disabilities suffered the closure of the rehabilitation services during the pandemic and the shortage of mobility assistive devices which were in line with what Mbazzi et al. (2022) and Rathore and Qureshi (2021) found. This was explained as having a negative effect on their recovery and might induce health complications (Rathore and Qureshi, 2021). A similar concern was shared by people with hearing disabilities facing a shortage of hearing aids and batteries, due to the closure of centers that usually provide them, in addition to the unaffordable cost of the hearing aids. This was in accordance with a Jordanian study that discussed the challenges faced by hearing aids users during the pandemic (Humanity and Inclusion, 2020). Finally, the closure of daycare centers for persons with severe intellectual disabilities was considered a burden experienced by our participants’ mothers of people with intellectual disabilities. Caring for persons with intellectual disabilities and the absence of professional support can affect the mental health and well-being of carers as has also been underlined by Courtenay and Perera (2020) and Patel et al. (2021).

In addition to the different access challenges PWDs were struggling with, the study heighted the devastating effect of COVID-19 on the financial situation of PWDs. Many were living in poverty prior to the pandemic. The stipends the PWDs usually obtain from the government were not provided in a timely manner and were insufficient. Palestinian PWDs were excluded from the governmental aid related to COVID-19 as explained by Diba and Zakaria (2020) and Banks et al. (2021). Also, the informal aid they used to receive stopped during the pandemic due to overall financial difficulties people faced.

To conclude, our study confirmed that COVID-19 measures undertaken by the Palestinian Authority in our case, as well as by other governments in LMICs, such as Bangladesh and Pakistan (Diba and Zakaria, 2020; Rathore and Qureshi, 2021), added on to the pre-existing inequalities and marginalization of PWDs. Furthermore, PWDs were also not represented in the government response plans and were left at the margin.

While most of the access difficulties shared by our study participants apply to many other countries, some challenges are context specific. While internal Palestinian reform is possible despite context, reform will continue to be limited in the face of ongoing Israeli military occupation of Palestinian land, depriving Palestinians from their basic rights, the Israeli control of so much of West Bank land and resources, as well as the restrictions imposed on the movement of people and goods by the Israeli army (Hamamra et al., 2021b).

This study indicates the need for the implementation of equitable policies with PWDs on top of the list of those needing assistance. However, until Palestinians can rule in freedom, sovereignty, self-determination, and of course political justice, it will be difficult for the PA to implement such policies.

## Data availability statement

The original contributions presented in this study are included in the article/supplementary material, further inquiries can be directed to the corresponding author/s.

## Ethics statement

The studies involving human participants were reviewed and approved by Institute of Community and Public Health, Birzeit University’s Research Ethics Committee. Written informed consent for participation was not required for this study in accordance with the national legislation and the institutional requirements.

## Author contributions

RG conceptualized the research idea, designed the study, and finalized the article. DK contributed to the conceptualization of the research, read and commented on the draft manuscript. SM conducted the field work, analyzed the transcripts, and wrote the draft manuscript. LA-S conducted the field work, analyzed the transcripts, and participated in the writing of the draft manuscript. All authors read, and approved the submitted version.
